# Comparison of IDEXX SediVue Dx^®^ urine sediment analyzer to manual microscopy for detection of casts in canine urine

**DOI:** 10.1111/jvim.16090

**Published:** 2021-03-24

**Authors:** Demitria M. Vasilatis, Larry D. Cowgill, Giosi Farace, Sarah Peterson, Murthy Yerramilli, Sean D. Owens

**Affiliations:** ^1^ Department of Pathology, Microbiology, and Immunology University of California, School of Veterinary Medicine Davis California USA; ^2^ Department of Medicine and Epidemiology University of California, School of Veterinary Medicine Davis California USA; ^3^ IDEXX Laboratories, Inc. Westbrook Maine USA

**Keywords:** automated analyzer, dog, prevalence, urinalysis, urine sediment

## Abstract

**Background:**

Detection of urinary casts is difficult due to their intermittent presence and deterioration in urine samples.

**Objective:**

To compare the performance of the IDEXX SediVue Dx^®^ Urine Sediment Analyzer (SediVue) with manual microscopy for the detection of urinary casts. We hypothesized that the SediVue analyzer would perform similarly to manual microscopy in cast detection.

**Animals:**

Four hundred forty‐three samples from 420 dogs from a hospital population.

**Methods:**

This is a prospective, cross‐sectional study. For SediVue analysis (software version [SW] 1.0.1.3), uncentrifuged urine was pipetted into a disposable cartridge. Seventy images were captured and processed by an onboard algorithm. For manual microscopy, urine was centrifuged to obtain sediment. Any cast identified by either method was considered a positive result (>0/low‐power field [LPF]). SediVue images were evaluated if casts were detected by either methodology. A revised sensitivity and specificity were calculated after image review and when using a threshold of >1 cast/LPF.

**Results:**

The sensitivity of the SediVue analysis for the detection of urinary casts was 53.7% (43.85%‐63.35%), and specificity was 86.0% (81.78%‐89.51%). After image review, the revised sensitivity/specificity was 52.0% (42.89%‐61.02%) and 90.6% (86.81%‐93.54%), respectively. When using a more clinically relevant threshold of >1/LPF, the sensitivity was 52.6% (35.82%‐69.02%) and specificity was 99.3% (97.85%‐99.85%).

**Conclusions and Clinical Importance:**

The SediVue provides moderate agreement to manual methodology for detection of casts in urine.

**Abbreviations**
CLS: clinical laboratory scientist
CNN: convoluted neural network
HPF: high ‐power field
HYA: hyaline cast
LPF: low ‐power field
NSOR: none seen or rare
PAT: non‐hyaline cast
RBC: red blood cell
SN: sensitivity
SP: specificity
VMTH: Veterinary Medical Teaching Hospital
WBC: white blood cell

## 1 INTRODUCTION

Urinary casts are concretions of mucoprotein or cellular elements (intact or breakdown products) that form in the distal renal tubular lumens and that are shed intermittently in the urine.^1^ Casts are often observed in humans with concentrated urine, acidic urine, or under conditions of low urine flow but can also be observed in urine samples from humans with primary renal disease or systemic disease that causes secondary renal disease.^2^ In the absence of a disease process, 1 to 2 casts/low‐power field (LPF/10× microscope objective) can be seen in clinically normal dogs and cats.^3^ Casts provide valuable clinical information about kidney health at low cost and with minimal invasiveness, although their detection can be problematic.^4^

Urinary casts are prone to physical deterioration, especially in alkaline, dilute, or stored urine.^3^, ^5^ Immediate (<1 hour after collection) analysis of fresh urine specimens is critical for their discovery and evaluation, yet routine urine assessment is often delayed or sent to reference laboratories for analysis. Moreover, there is high interobserver variability and inaccuracy with regard to identifying casts in human urine sediment.^6^, ^7^ It has been speculated unfamiliarity, and lack of training in urine sediment examination by veterinary technicians and veterinarians might decrease accurate identification of formed elements including casts.^8^ For instance, manipulation of light alignment on the microscope is critical for the identification of formed elements, including casts, which might not always be implemented in veterinary practice.

Several instruments have been introduced for use in the clinical evaluation of human urine in an attempt to automate urine sediment examination and minimize these challenges. These laboratory analyzers bypass the issues that might occur due to sample shipment and offer the potential of increased intra‐assay precision, decreased turnaround time, and use of a smaller sample volume than manual urine sediment examination.^9^, ^10^, ^11^, ^12^ However, these analyzers have low sensitivity and high specificity for cast detection when compared to manual examination of urine sediment^12^, ^13^ and have been considered unreliable in some studies.^14^

The SediVue Dx^®^ (SediVue) was the first automated urine analyzer marketed for use in veterinary species. The instrument images approximately 45 high‐power fields (HPF/40× microscope objective) of urine sediment, which are then analyzed with image recognition algorithms adapted for dog and cat urine. A study reported that the SediVue analyzer had good agreement with manual sediment examination for most formed elements, including red blood cells (RBC), white blood cells (WBC), and various crystals; however, casts were not evaluated.^8^

The objective of this study was to compare the SediVue analyzer with manual sediment examination (i.e., “gold standard” or “reference method”) for detection of urinary casts in dog urine samples. We hypothesized that the analyzer would perform similarly to instruments used in human medicine providing low sensitivity and high specificity for cast detection.

## 2 MATERIALS AND METHODS

### 2.1 Study design and sample selection

This was a prospective, cross‐sectional study. Excess urine obtained between June 2018 and November 2018 from 443 routine urinalyses from 420 client‐owned dogs that presented to the William R. Pritchard Veterinary Medical Teaching Hospital at the University of California, Davis (VMTH), was used. Samples were included if a volume of ≥1.0 mL was present to obtain sediment for manual examination, and at least 165 μL of the uncentrifuged sample could be spared for SediVue analysis. Urine from healthy or ill dogs of all ages, sexes, and breeds were accepted. Multiple samples from the same dog were permitted if >24 hours passed between sample submissions. Samples were excluded if they had less than 1.0 mL of volume, had gross environmental or fecal contamination, or if the sample required dilution for SediVue processing. Each sample was examined by manual microscopy and by the SediVue in tandem.

### 2.2 Manual sediment examination

Each urine sample was examined within 30 minutes of collection by a licensed clinical laboratory scientist (CLS). Seven CLS personnel were involved in the study. Well‐mixed samples were centrifuged at 360*g* for 6 minutes (ALC Centrifuge PK 110). Excess supernatant was decanted until 0.2 to 0.5 mL remained to resuspend the pellet. One drop (~10 μL) of resuspension was placed on a glass slide and covered with a glass coverslip (22 mm × 22 mm). The entire slide was examined using light microscopy at low power (LPF/10× microscope objective), with attention paid to the coverslip edges for casts. A minimum of 10 high‐power fields (HPF/40× microscope objective) were examined by both light and phase contrast microscopy. The CLS then assigned casts to a semiquantitative category per LPF examined: “none”‐ none seen; “rare”‐ not present in every field observed; “few”‐ each field contains a small number (1‐2 casts/LPF); “mod‐ each field contains some but not packed (3‐5 casts/LPF); “many”‐ each field contains many casts (>5/LPF). Casts were recorded as hyaline, granular, cellular, waxy, or other.

### 2.3 SediVue analysis

Urine sediment examination by the SediVue analyzer was performed in tandem with manual microscopy, with 165 μL of well‐mixed, uncentrifuged urine manually pipetted into a disposable cartridge by a CLS, while the remaining urine sample was centrifuged for manual microscopy. The CLS was then blinded from the analyzer results. After SediVue centrifugation (30 seconds, 260*g*), 70 images of the sediment were captured by a camera within the instrument. According to the instrument's manufacturer, these 70 images represent the equivalent of approximately 45 HPF and represent approximately 10 μL of the total 165 μL pipetted into the cartridge. An internal computer system using a convolutional neural network (CNN) (SW 1.0.1.3) algorithm processed the images and identified and quantified any casts present at HPF. The quantification was then converted into number of casts per LPF. Casts were identified as either hyaline or non‐hyaline. Non‐hyaline casts encompass all casts that are not strictly hyaline (e.g., granular, cellular, waxy, mixed). Cast types were reported as a semiquantitative result: none to rare (NSOR); suspect presence; and >1/LPF. The SediVue analyzer used for this study was in “research mode,” which allowed for the additional reporting of casts per HPF in addition to the semiquantified reporting. This HPF reporting was used to detect samples that had rare casts that would otherwise not be reported by this software version (SW 1.0.1.3) in terms of LPF, as this particular version did not create an LPF extrapolation for “none to rare” and “suspect presence” specimens. All other hardware and software features were identical to that used in clinical practice.

Macroscopic appearance (i.e., color and clarity) and other formed elements found on sediment examination (e.g., WBC, RBC, crystals, epithelial cells, sperm, bacteria, mucus, lipid) were evaluated and recorded for each sample. Macroscopic appearance was subjectively determined by the CLS evaluator. Other formed elements on sediment examination were counted per HPF. Reference intervals for WBC (<3/HPF) and RBC (<5/HPF) were used. Crystals and epithelial cells were recorded per HPF and are described here as “present” if >1/HPF. Bacteria, sperm, and mucus strands were recorded semiquantitatively (none, rare, few, moderate, many) and are described here as “present” if “few,” “moderate,” or “many” were observed. Lipid was described as either “present” or “absent.”

### 2.4 SediVue image analysis

Visual analysis of the images captured by the SediVue for all samples considered positive for casts by either methodology (true‐positive, false‐negative, and false‐positive results), and 50 random samples considered negative by both methodologies (true‐negative results) was performed by a board‐certified veterinary clinical pathologist (SDO) and clinical pathology resident trainee (DMV). A third board‐certified veterinary clinical pathologist was consulted if there was disagreement between the 2 reviewers to reach a consensus.

### 2.5 Statistical analysis and thresholds

Statistical analysis was performed using open‐access online software (MedCalc Statistical Software version 16.4.3 (MedCalc Software, Ostend, Belgium [https://www.medcalc.org; 2016]) and commercially available software programs (Microsoft Excel 2019; Microsoft Corporation, Redmond, Washington). Overall sensitivity (Sn) and specificity (Sp) of the SediVue compared to manual microscopic examination was determined using a threshold of >0/LPF as a positive result (ie, any sample positive for casts). With >0/LPF as a threshold, positive results were those semiquantitatively reported as “rare,” “few,” “mod,” and “many” by manual microscopy, and reported as “NSOR” if any casts were detected per HPF, “suspected presence,” and “>1/LPF” by the SediVue. Negative results were those with no casts present on manual microscopy (ie, semiquantitatively reported as “none”) and SediVue samples reported as “NSOR” if no casts were detected per HPF. Quantification of casts per HPF was exclusively available in the “research mode” version of the SediVue software as previously described.

Additional sensitivity and specificity values were calculated using a modified threshold of ≥1/LPF for a positive result and <1/LPF for a negative result. Samples semiquantitatively described as having “none” or “rare” casts present by manual methodology, and all samples reported as “NSOR” or “suspect presence” by the SediVue were considered negative results when the modified threshold was used. Samples semiquantitatively described as “few,” “moderate,” or “many” casts were considered a positive result by manual methodology, and samples that reported “>1/LPF” were considered a positive result by the SediVue.

The following scale was used to rate sensitivity and specificity, which was previously used by Hernandez et al. for other SediVue parameter assessments: excellent (95.0%‐100.0%), good (85.0%‐94.9%), moderate (70.0%‐84.9%), fair (60.0%‐69.0%), and poor (≤59.9%).^8^ Cohen's kappa coefficient was calculated to determine agreement between both methodologies. The scale used to classify Cohen's kappa coefficient was as follows: excellent (0.81‐1.00), substantial (0.61‐0.80), moderate (0.41‐0.60), fair (0.21‐0.40), and slight (0.0‐0.20).^15^

## 3 RESULTS

### 3.1 Samples

Four hundred fifty‐five urine samples were examined of which 12 samples were excluded as they required dilution for SediVue processing, which was not performed. Because the CLS operator was blinded from the analyzer results section where analyzer prompting for dilution is stored, these samples were not diluted and were consequently excluded as they are not acceptable results per the analyzer's instructions. Ultimately, 443 urine samples from 420 dogs were included in the study. The average total volume of submitted urine was 4.6 mL (range of 1.0‐5.0 mL), with 2.5% (11/443) between 1.0 and 2.0 mL, 5.0% (22/443) between 2.1 and 3.0 mL, 15.1% (67/443) between 3.1 and 4.0 mL, and 77.4% (343/443) between 4.1 and 5.0 mL. Macroscopic appearance of all samples (n = 443) was recorded. Sample color was recorded as: colorless (1/443; 0.2%), straw (11/443; 2.5%), yellow (409/443; 92.3%), dark yellow (10/443; 2.3%), amber (10/443; 2.3%), red (1/443; 0.2%), or brown (1/443; 0.2%). Sample clarity was recorded as clear (52/443; 11.7%), slightly hazy (233/443; 50.3%), hazy (86/443; 19.4%), cloudy (69/443; 15.6%), or opaque (3/443; 0.7%). Moreover, other formed elements for all samples were recorded in addition to casts (eg, WBC, RBC, crystals, epithelial cells, sperm, bacteria, mucus, lipid). Ninety‐eight samples (98/443; 22.1%) had hematuria and/or pyuria (ie, “an active sediment”); 75 of 443 (16.9%) samples had crystalluria; 397 of 443 (89.6%) samples had epithelial cells present; 15 of 443 (3.4%) samples had sperm present; 38 of 443 (8.6%) samples had bacteria present; 342 of 443 (77.2%) had mucus present; and 144 of 443 (32.5%) had lipid droplets present (Table 2). Macroscopic results have been organized in Table 2 as “normal” or “abnormal” to reflect the typical color (colorless‐amber) and clarity (clear‐slightly hazy) of urine from normal healthy dogs.^16^

There were 108 samples with casts identified by manual microscopy, establishing an overall prevalence of 24.4% (108/443), with a 4.0% (18/443) prevalence of non‐hyaline casts in this population. Three hundred thirty‐five samples were negative on manual microscopy. Of the 108 samples positive for casts by manual microscopy, 68.5% (74/108) had “rare” casts present, 23.1% (25/108) had “few” casts present, 7.4% (8/108) had “moderate” numbers of casts present, and 0.9% (1/108) had “many” casts present. Of the 105 samples reported positive for casts by SediVue analysis, 61.0% (64/105) had only “rare” (NSOR) casts detected, 17.1% (18/105) had “suspected” casts detected (suspect presence), and 21.9% (23/105) had >1/LPF detected. Three hundred thirty‐eight samples were reported negative by the SediVue (0/HPF).

### 3.2 Comparison of SediVue to manual microscopy in detection of casts

Overall, 102 of 108 (94.4%) positive samples on manual microscopy had hyaline casts; 90 of 108 (83.3%) samples had hyaline casts only; and 12 of 108 (11.1%) samples had both hyaline and nonhyaline casts. Eighteen of 108 (16.7%) positive samples on manual microscopy had non‐hyaline casts, and 6 of 108 (5.6%) samples had non‐hyaline casts only. All non‐hyaline cast samples (18/18 [100%]) contained granular casts; 2 samples (2/18 [11.1%]) also contained cellular (epithelial) casts, and 1 sample (1/18 [5.6%]) also contained white blood cell casts. Distinction between different variations of granular casts (fine vs. coarse) was not recorded.

For the SediVue analysis, the number of samples reported positive for casts was 105 of 443 (23.7%) samples. Overall, 47 of 105 (44.8%) samples had reported hyaline casts and 79 of 105 (75.2%) samples had reported non‐hyaline casts; 26 of 105 (24.8%) samples had reported hyaline casts only; 58 of 105 (55.2%) samples had reported non‐hyaline casts only; and 21 of 105 (20.0%) samples had reported both. Distinction was not made between the types of non‐hyaline casts by the SediVue analysis.

When compared to manual microscopy for the detection of any casts present in the samples (>0/LPF), the SediVue had an initial sensitivity of 53.7% and initial specificity of 86.0% (Table 1). Cohen's kappa coefficient was calculated (*κ* = 0.40) and determined moderate agreement between the methods (Table 1). The SediVue made 47 false positives and 50 false negatives prior to image review (Table 3).

**TABLE 1:** Sensitivities and specificities of the SediVue in comparison to manual microscopy for the detection of casts

Threshold	Sensitivity (95% CI)	Specificity (95% CI)	Kappa (95% CI)
>0/LPF			
Before review	53.7% (43.85%‐63.35%)	86.0% (81.78%‐89.51%)	*κ* = 0.40 (0.30‐0.50)
After review	52.0% (42.89%‐61.02%)	90.6% (86.81%‐93.54%)	
≥1/LPF			
Before review	47.1% (29.78%‐64.87%)	98.3% (96.51%‐99.31%)	*κ* = 0.53 (0.37‐0.69)
After review	52.6% (35.82%‐69.02%)	99.3% (97.85%–99.85%)	

*Note:* Sensitivity and specificity were calculated before and after image review for both thresholds evaluated (>0/LPF and >1/LPF).

Abbreviations: CI, confidence interval; LPF, low‐power field.

### 3.3 Manual review of SediVue images

Images for all samples deemed positive for casts by either methodology were re‐examined to determine the accuracy of the SediVue's false‐negative and false‐positive results. In total, images from 155 samples were reviewed. This review included the following: 58 samples both methodologies considered positive for casts (true positives), 47 samples only the SediVue considered positive for casts (false positives), and 50 samples the manual methodology considered positive for casts but the SediVue did not (false negatives) (Table 3). Of these false‐negative samples, 70.0% (35/50) had no casts present in the images and 30.0% (15/50) had casts present in the images that the SediVue algorithm did not detect. Macroscopic appearance and other formed elements for false‐negative and false‐positive results are outlined in Table 2.

**TABLE 2:** Macroscopic appearance and other formed elements reported on sediment examination for all urine samples evaluated

	Color		Clarity	
(A)	Normal	Abnormal	Normal	Abnormal
All (n = 443)	441/443 (99.5%)	2/443 (0.5%)	285/443 (64.3%)	158/443 (35.7%)
FP (n = 47)	47/47 (100.0%)	0/47 (0.0%)	30/47 (63.8%)	17/47 (36.2%)
FN (n = 50)	49/50 (98.0%)	1/50 (2.0%)	35/50 (70.0%)	15/50 (30.0%)

	Other formed elements						
(B)	WBC±RBC	CRY	EPI	MUC	BAC	SPM	LIP
All (n = 443)	98/443 (22.1%)	75/443 (16.9%)	397/443 (89.6%)	342/443 (77.2%)	38/443 (8.6%)	15/443 (3.4%)	144/443 (32.5%)
FP (n = 47)	10/47 (21.3%)	10/47 (21.3%)	42/47 (89.4%)	41/47 (87.2%)	3/47 (6.4%)	1/47 (2.1%)	38/47 (80.9%)
FN (n = 50)	17/50 (34.0%)	11/50 (22.0%)	46/50 (92.0%)	47/50 (94.0%)	4/50 (8.0%)	2/50 (4.0%)	42/50 (84.0%)

*Note:* Samples deemed as false positives or false negatives prior to image review are delineated. A, Normal macroscopic color includes colorless and straw‐, yellow‐, dark yellow‐, and amber‐colored samples and abnormal includes brown‐ and red‐colored samples. Normal macroscopic clarity includes clear and slightly hazy samples, and abnormal includes hazy, cloudy, and opaque samples. B, Other formed elements reported on sediment examination for all samples. Samples with white blood cells or red blood cells are considered to have an “active sediment.”

Abbreviations: BAC, bacteria; CRY, crystals; EPI, epithelial cells; FN, false negatives; FP, false positives; LIP, lipid; MUC, mucus; RBC, red blood cell; SPM, sperm; WBC, white blood cell.

**FIGURE 1:**
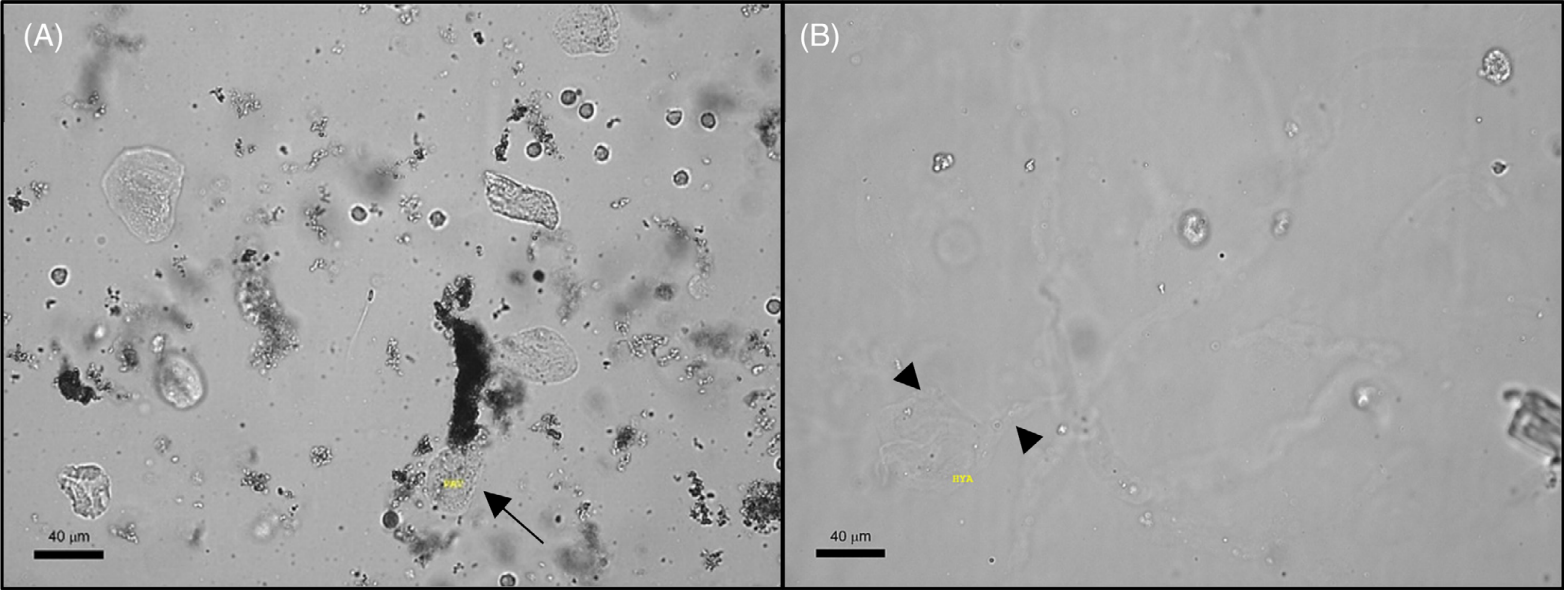
Examples of inaccuracies in the identification of casts by the SediVue. A, Misidentification of squamous cells for non‐hyaline casts (arrow). B, Mucus strands are observed out of focal plane and within focal plane and misidentified as hyaline casts (arrowheads). Squamous cells and mucus strands were commonly misidentified as casts in false‐positive results. Each image represents approximately 66% of a 40× objective high‐power field. Scale bar = 40 μM. Abbreviations: HYA, hyaline cast; PAT, non‐hyaline cast

Among the 47 false‐positive results, 63.8% (30/47) of the samples had other formed elements misidentified as casts by the SediVue (eg, squamous epithelial cells, mucus strands) (Figure 1). However, 36.2% (17/47) of these false‐positive results had in fact identified a cast that was not observed during manual microscopy (5 hyaline casts, 14 non‐hyaline casts), making them true positives. Of the original 58 true positives, the SediVue misidentified mucus and debris as a cast in 10 samples, and with no real casts present in these images, the samples were recategorized as false negatives (Table 3). Finally, images from 50 random samples of the 288 samples considered negative by both methodologies (true negatives) were also reviewed, and all images reviewed were negative for casts. With these image review findings, a revised sensitivity of 52.0% and revised specificity of 90.6% were determined (Table 1).

**TABLE 3:** Results before and after SediVue image review for threshold of >0/LPF and modified threshold of ≥1/LPF

Threshold	True positives	True negatives	False negatives	False positives
>0/LPF				
Before review (initial)	58	288	50	47
Casts subtracted	10	N/A	N/A	17
Casts added	17	0	10	N/A
After review (revised)	65	288	60	30
≥1/LPF				
Before review (initial)	16	402	18	7
Casts subtracted	N/A	N/A	N/A	4
Casts added	4	N/A	N/A	N/A
After review (revised)	20	402	18	3

*Note:* For >0/LPF threshold, 10 true positives were recategorized to false negatives, and 17 false positives were recategorized as true positives after image review. For ≥1/LPF threshold, 4 false positives were recategorized to true positives after image review. This modified threshold excluded rare casts as a positive result.

Additionally, sensitivity and specificity were calculated based on the reviewed images and using a modified threshold of ≥1/LPF for a positive result. By manual methodology, 34 samples were positive (ie, semiquantitatively described as “few,” “moderate,” or “many”) and 409 samples were negative (ie, “none” or “rare” casts seen). Initially, there was 16 true positives, 18 false negatives, 7 false positives, and 402 true negatives when the SediVue was compared to manual methodology (Table 3). The initial sensitivity (47.1%) and specificity (98.3%) were calculated. Cohen's kappa coefficient was calculated (*κ* = 0.53) and determined moderate agreement between the methods when the modified threshold (≥1/LPF) was used (Table 1). After image review, there were 20 true positives, 18 false negatives, 3 false positives, and 402 true negatives. Ultimately, when a modified threshold of ≥1/LPF was used, the SediVue's revised sensitivity was 52.6% (20/38) and revised specificity was 99.3% (402/405) when compared to the manual method.

## 4 DISCUSSION

The SediVue is an automated urinalysis sediment analyzer for dog and cat samples. We compared the performance of the SediVue to manual microscopy for detecting urinary casts in fresh dog urine. The ability of the SediVue to detect casts was initially determined to be equivocal, with a low sensitivity (initial Sn = 53.7%) but a moderately high specificity (initial Sp = 86.0%), similar to human automated urine analyzers. After image review, the sensitivity did not significantly change, but the specificity improved (revised Sp = 90.6%). In samples where casts were in densities commonly considered clinically relevant, ≥1/LPF, the specificity was excellent (modified threshold Sp = 99.3%). Evaluation of the SediVue images revealed areas for analyzer improvement, as well as instances when the analyzer performed better than a human operator. Overall, the SediVue had moderate agreement with manual methodology (*κ* = 0.40).

**FIGURE 2:**
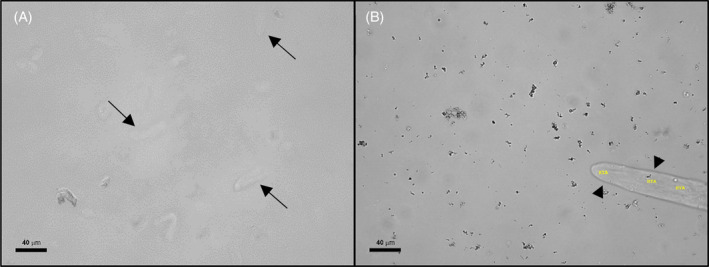
Images of unstained urine sediment containing casts out of the focal plane (A), and in the focal plane (B). A, Out‐of‐focus casts are identified with arrows. B, In‐focus cast is identified with arrowheads. The inability to detect casts out of the focal plane is considered a possibility for false‐negative results by the SediVue. Each image represents approximately 66% of a 40× objective HPF. Scale bar = 40 μM. Abbreviations: HYA, hyaline cast

Review of the images from the SediVue's false‐negative samples demonstrated that the majority of these misses (70.0%) were due to the absence of casts in the images. The combination of the small volume used by the SediVue analyzer and the low quantity of casts present in the majority of the samples (eg, 68.5% had “rare” casts present per manual methodology) are considered key reasons for the SediVue's false‐negative results and poor sensitivity (initial Sn = 53.7%, revised Sn = 52.0%). This could be remedied by increasing the volume of urine evaluated by the analyzer. Another limitation that might affect sensitivity is the analyzer's inability to focus through different planes of the sediment, which could have prevented the analyzer from detecting casts that were present in some specimens. Occasional out‐of‐focus or partly in focus casts were observed during manual review of the images, supporting this idea (Figure 2). Only fresh urine samples from dogs presenting to the VMTH were used, and urinalysis was performed within 30 minutes after collection. This is believed to have optimized cast recovery and therefore sensitivity for both methodologies by decreasing potential cast degradation because of storage.^5^ A smaller subset (30.0%) of the failed detections was due to algorithm error, which could be improved with CNN software revision.

Specificity for the SediVue was considered good (initial Sp = 86.0%, revised Sp = 90.6%). Image review from false‐positive samples (n = 47) revealed that the majority (63.8%) were mislabeled debris, environmental contamination, or mucus, and therefore specificity could be improved with CNN software modifications. Because of this misidentification of debris for casts, we recommend review of SediVue images as advised by the manufacturer and as other studies have also suggested regarding automated urine sediment analyzers, especially free‐catch specimens that are subject to urogenital contaminants.^9^, ^11^, ^13^ Upon image review, 36.2% of these false positives were in fact true positives missed by manual microscopy, thus highlighting the fallibility of the reference method. As such, a revised sensitivity and specificity were calculated to account for this discovery (revised Sn = 52.0%, revised Sp = 90.6%). One limitation of the study is that not all images from the true negatives were reviewed, but rather images from 50 random samples from the group were chosen to represent the subset. Therefore, additional false‐negative results by the reference method remain a possibility.

Macroscopic appearance and other formed elements on sediment examination for the false‐negative and false‐positive results were similar to the overall study population, with the exception of more samples containing mucus and lipid (Table 2). These elements, in conjunction with debris and squamous cells, were observed on image review and contributed to false‐positive results (Figure 1B). However, these elements also contributed to false negatives, as 10 of 58 true positives were relabeled as false negatives after image review because mucus and debris were misidentified as casts. As a result, algorithm software revision for accurate identification of mucus and lipid is recommended for improvement of cast identification by the SediVue. Additionally, false positives and false negatives also had slightly more samples containing crystals, and false negatives also had more samples with an active sediment when compared to the overall study population. Crystals, however, were not misidentified by the SediVue for casts on image review and are not a likely contributor to its false positives. The small increase (+11.9%) in false‐negative samples with an active sediment and with crystals (+5.1%) might be coincidental as the majority of false negatives are due to the absence of a cast in the urine aliquot run by the SediVue. In contrast, the false negatives made by the reference method (n = 17) might have occurred because of increased formed elements and denser sediment preparations, leading to obscured visualization of casts by the CLS. Further studies are needed to investigate these possibilities.

In this study, we set an initial cast threshold of >0/LPF as a positive result. However, the SediVue analyzer does not alert the operator that casts are present in the images until “suspected casts” or “>1 cast per LPF” are detected, which is based on a calculation from detections per HPF. We justified the use of >0/LPF because rare casts are described in routine urinalyses by sediment examination in practice and, as such, are part of a comprehensive methodology comparison study. Additionally, sometimes rare casts are also clinically relevant (eg, renal tubular cellular casts). Of the 105 samples reported positive for casts by SediVue analysis, 61.0% of these had rare casts present and would have been overlooked with the SediVue reporting criteria of “NSOR.” This could potentially lead to delayed diagnosis of systemic or renal diseases, but we recognize that this might also lead to overinterpretation.

In contrast, because 1 to 2 casts/LPF are sometimes observed in normal urine, we also analyzed the sensitivity and specificity when casts were ≥1/LPF (or semiquantitatively described as “few,” “moderate,” or “many” by manual methodology) to reflect potentially more clinically relevant samples. The sensitivity of the SediVue did not change with the modified threshold (initial revised Sn = 52.0% vs modified threshold Sn = 52.6%), but specificity became excellent (initial revised Sp = 90.6% vs modified threshold Sp = 99.3%). One reason for the improvement in specificity is that the modified threshold classified samples with “rare” casts as true‐negative results, which is the majority of samples in the study. Nevertheless, this improvement in specificity demonstrates that the SediVue had few false‐positive results claiming casts were present in numbers >1/LPF.

There are a few possible reasons as to why the SediVue analyzer detected more casts than manual microscopy. First, manual microscopy was performed by multiple CLS without confirmation by an additional observer, and consequently, sediment preparation and interpretation could have been subject to error. Confidence in the reference method could have been improved with verification by a second operator or with photomicrographs taken of the evaluated fields. There was no standard pattern for selection of fields by manual microscopy that was applied across observers. Additionally, the greater number of HPFs examined by the SediVue could have led to increased detections in these cases, although, not enough to improve the overall sensitivity. The idea that the SediVue has a “gentler” centrifugation process leading to increased preservation of formed elements and detection has been proposed,^17^ but this seems unlikely, as the g‐force used for manual urinalysis (360*g*) was similar to the g‐force used in the SediVue analyzer (260*g*) in this study. However, the longer centrifugation time for the manual method (6 minutes) might have had a negative impact on cast recovery and can be considered for a future study.

Manual microscopy is the gold standard for urine sediment evaluation and was used as the reference standard for the SediVue's sensitivity and specificity calculations for casts. Limitations of manual microscopy include imprecision due to inadequate standardization of laboratory techniques (eg, variable sample volume, variable volume used for pellet resuspension), preparation technique, interobserver variability, sample propensity for artifacts, and cost and labor requirements.^8^, ^9^, ^10^, ^11^, ^12^, ^13^, ^14^ These limitations of manual microscopy were also limitations in our study. Urinalyses were performed by 7 licensed CLS, which leaves the gold standard results subject to interobserver variation. Interobserver and intraobserver SediVue precision was not evaluated in this study. However, in a recent human study, agreement between nephrologists for urinary cast identification was 59%,^18^ supporting the idea that the reference method is prone to interobserver imprecision and unreliability as the gold standard. Because the SediVue and manual sediment exam were performed in tandem, no sample remained to evaluate the precision of the SediVue, and no aliquot of urine was partitioned off beforehand to perform this task.

Interobserver variation was minimized during SediVue image review by using 2 human evaluators and a third evaluator when needed to arrive at a majority decision. Human identification of casts on the images sometimes proved challenging because images were only available at HPF. This truncated the cast of interest at times, creating uncertainty about its identity. Moreover, total length and overall size of the cast was often difficult or impossible to determine because of the image magnification. These challenges could have led to unintentional misidentification of casts by the observers. This is also viewed as a possible limitation for the analyzer, as it might have contributed to the false‐positive results.

This study was performed at a tertiary‐care veterinary institution, and case bias might have led to more casts present in urine than anticipated in general veterinary practice. To the author's knowledge, there is no information on the prevalence of casts for a heterogeneous population of dogs, although prevalence of casts in dogs with renal disease has been discussed.^19^ The overall prevalence of casts in this population was 24.4% (108/443), with 4.0% (18/443) prevalence of non‐hyaline casts and 23.0% (102/443) prevalence of hyaline casts. With additional detection of casts by the analyzer, overall prevalence could be revised to 28.2% (125/443), with a revised prevalence of 7.2% (32/443) and 24.2% (107/443) for non‐hyaline and hyaline casts, respectively. Another important consideration is that urine sediment examination was performed within 30 minutes of collection by a trained, licensed CLS and may not be a practicable standard in most community general practices.

Overall, the SediVue had good specificity for cast identification (>0/LPF), but excellent specificity for cast identification when using a more clinically relevant threshold (>1/LPF); however, sensitivity in this study was poor. We postulate that the low sample volume accepted by the SediVue and the low density of casts in the majority of samples could be major contributors to the analyzer's sensitivity. In the future, SediVue images could be compared to photomicrographs taken of the sediment during manual microscopy to improve performance and agreement between the methods. Additionally, improvements to the CNN software to distinguish between cellular debris and mucus could improve the specificity. The SediVue had moderate agreement with manual methodology when performed by a licensed CLS and may provide an option for a rapid initial assessment of the urine sediment, replacing the need for complete sediment examination in some clinical samples. Future studies would also be needed to evaluate the performance of the SediVue compared to veterinary technicians working within a general practice environment.

## CONFLICT OF INTEREST DECLARATION

G. Farace, S. Peterson, and M. Yerramilli are employed by and have stock or stock options with IDEXX Laboratories, Inc. L. Cowgill has received travel reimbursement and speaker honoraria from IDEXX Laboratories, Inc. within the past 5 years.

## OFF‐LABEL ANTIMICROBIAL DECLARATION

Authors declare no off‐label use of antimicrobials.

## INSTITUTIONAL ANIMAL CARE AND USE COMMITTEE (IACUC) OR OTHER APPROVAL DECLARATION

Authors declare no IACUC or other approval was needed.

## HUMAN ETHICS APPROVAL DECLARATION

Authors declare human ethics approval was not needed for this study.
